# International medical electives during and after the COVID-19 pandemic - current state and future scenarios: a narrative review

**DOI:** 10.1186/s12992-022-00838-0

**Published:** 2022-04-22

**Authors:** Maximilian Andreas Storz

**Affiliations:** Centre for Complementary Medicine, Department of Internal Medicine II, Faculty of Medicine, University of Freiburg, 79106 Freiburg, Germany

**Keywords:** Abroad elective, International medical elective, Oversea Elective, Medical education, Learning, Global Health, SARS-CoV-2 Pandemic, COVID-19

## Abstract

**Background:**

International medical electives are an important and popular component of the academic curriculum in many medical schools and universities worldwide. The purpose of abroad electives is to provide medical students with an opportunity to gain a better understanding of education and healthcare in an international context. The COVID-19 pandemic, however, has substantially changed the international elective landscape. Travel restrictions, closures of international elective programs and the expansion of virtual methods for education caused a widespread disruption to abroad electives. A comprehensive analysis with regard to other consequences for abroad electives, however, has not been done before. Thus, we sought to a) summarize the current transformation of the international medical elective and b) to address potential challenges for post-pandemic international medical electives.

**Methods:**

The methodology employed is a multidisciplinary narrative review of the published and grey literature on international electives during the last two years of the COVID-19 pandemic.

**Results:**

Students worldwide had electives postponed or canceled. Apart from evident immediate pandemic-related consequences (such as the substantial decline in global electives and impaired elective research opportunities for educators), there are other several problems that have received little attention during the last two years. These include challenges in the elective application process, poorly-understood consequences for host institutions, and growing global (ethical) disparities that are likely to increase once elective programs will gradually re-open. There is ample evidence that the post-pandemic elective landscape will be characterized by increasing elective fees, and a more competitive seat-to-applicant ratio. Ethical problems for international electives arising from an unequal global vaccine distribution will pose an additional challenge to students and elective coordinators alike.

**Conclusion:**

The COVID-19 pandemic transformed the international medical elective landscape in an unprecedented way, and future generations of medical students will face a series of additional challenges when applying for global medical electives.

## Introduction

International medical electives are an important component of the academic curriculum in medical schools and universities throughout the globe [1]. While the number of students undertaking abroad electives depends on country, time and curriculum structures, studies reported that up to 50% of students in high income countries may partake in international electives [2, 3]. For many medical students, abroad electives are the first encounter to global health and a valuable learning experience [4, 5]. The purpose of abroad electives is to provide medical students with an opportunity to gain a better understanding of healthcare and medical education in an international context [6, 7]. The emphasis on many abroad electives is on global and public health, prevention and primary care [7].

In most cases, international medical electives are clinical immersion experiences, with student contributions ranging from passive observation (also termed shadowing) to active involvement in multiple aspects of patient care (including but not limited to clinical assessment, case management, and participation in invasive procedures –under varying degrees of supervision) [8].

**Fig. 1:**
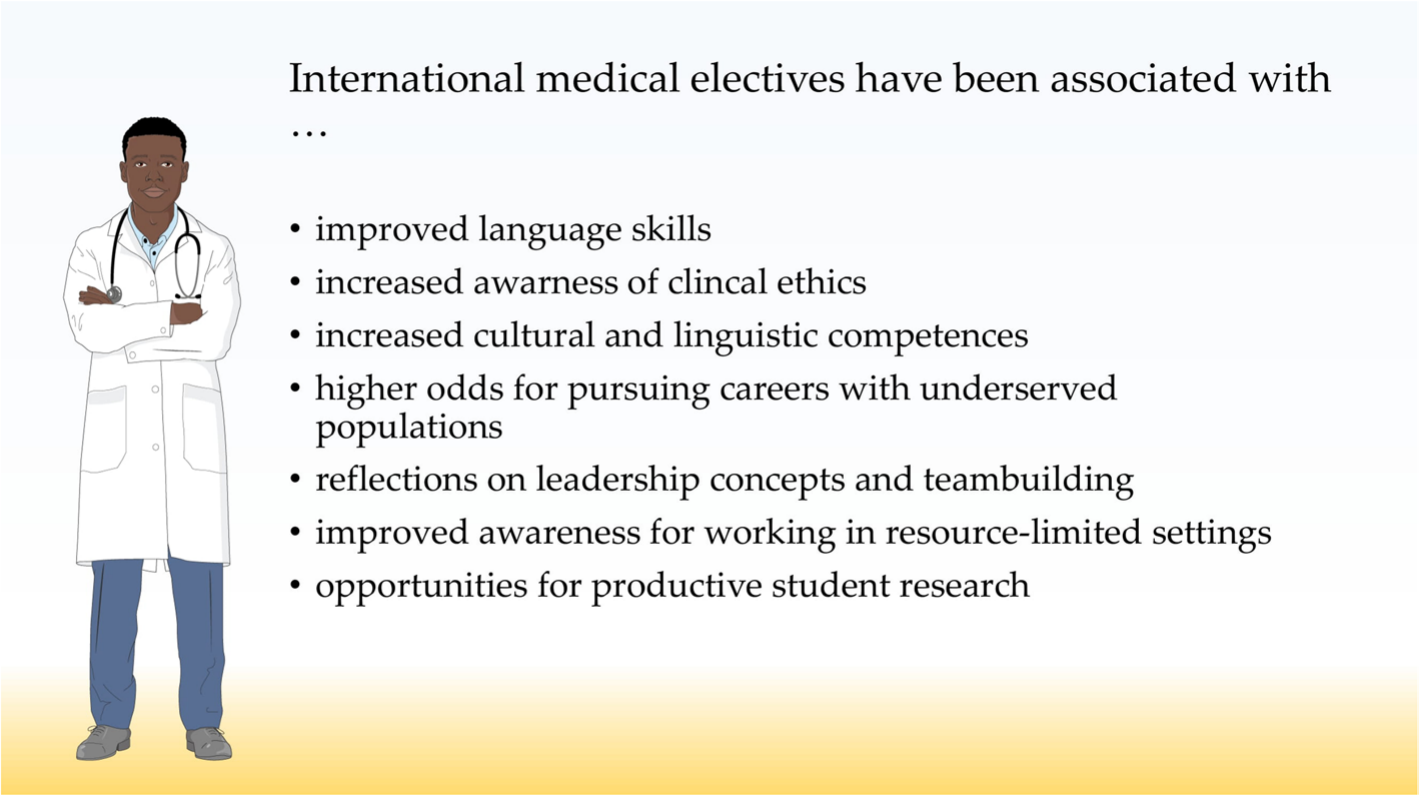
Merits associated with international medical electives [11–18]. Modified from Servier Medical Art database by Servier (Creative Commons 3.0)

Notably, international electives have been associated with various educational benefits. Several authors emphasized that abroad electives may promote reflective self-relativisation, personal growth, and help enhancing the performance of undergraduate medical students [1, 6, 9]. In addition to that, abroad electives contribute to medical professional identity formation and increase students’ interest in humanitarian efforts and volunteerism [9, 10]. Benefits and assets associated with abroad medical electives in previous studies are shown in Fig. 1 [11–18].

Motivations to partake in electives may depend on a student’s origin and cultural background. Opportunities to experience different healthcare systems and resource-different settings are the main drivers for both students from high- and low income countries [19]. Students from low income countries often reported that their elective experience positively affected their chances of pursuing training abroad (for example in the United States) and enhanced their professional development [19]. On the other hand, students from high income countries emphasized exposure to different cultures and languages. With the advent of COVID-19, however, continuing education changed abruptly for both groups [20].

To minimize the risk of virus transmission, virtual telehealth options rapidly expanded. Healthcare spendings (which are significantly associated with health outcomes [21]), increased in many countries, while healthcare utilization often decreased at the same time. Governments invested in healthcare infrastructure, personal protective equipment and medical devices [22]. Notably, these implemented public health interventions that aimed to contain the disease also affected healthcare personnel and employees [23]. In the clinical care setting, the pandemic has led to a complete paradigm shift in the mode of instruction [23, 24].

In-person training has been frequently cancelled in favor of virtual forms of pedagogy, and final year students—who were due to complete their rotations and sit for their final examination—were hit hard in particular [23, 24]. While several authors illuminated the effects of the pandemic on medical education in general [25–29], little is known about the consequences and implications for international medical electives in the global context.

The present study sought to address this gap in the literature. To allow for a better understanding of the repercussions of the pandemic on abroad electives, we performed a literature research investigating the current transformation of the international medical elective and aiming to address potential challenges for post-pandemic abroad medical electives.

### Methodology

This narrative review synthesizes the literature on the potential impacts of the COVID-19 pandemic on international medical electives. It is based on a PubMed and Google Scholar literature research using various combinations of the following search terms: “COVID-19”, “Coronavirus”, “SARS-CoV-2”, International Elective, Abroad Elective, Medical Elective, Medical Education. The literature research was performed in January 2022 by the author of this article (MAS). Original articles, short reports, reviews, commentaries and letters to the editor were included in this review. To increase the number of potentially eligible studies, we manually screened reference lists of the included articles to ensure that all relevant publications were identified. To identify additional publications, we also used Google Scholar’s “cited by” function. In addition to that, we contacted several renowned experts in the field by e-mail.

Articles were included if they reported effects of the COVID-19 pandemic on abroad electives, irrespective of country, setting (low or high income country) or population (both undergraduate and graduate students were considered). Studies were included irrespective of their outcome, as we explored both positive and negative effects related to COVID-19. For this review, we considered only English and German language articles. Pre-pandemic studies and articles (prior to October 2019) were not considered except when they provided important background information for international electives in general.

In light of the generally very limited literature with regard to international electives, a narrative review was deemed most appropriate as it provides a comprehensive overview of COVID-19-related consequences and wider literature contributing to this specific area, incorporating a diverse range of sources and article types.

### International electives during the COVID-19 pandemic (2020 – 2022)

The COVID-19 pandemic has substantially changed global health education and the international elective landscape [30, 31]. The pandemic put pressure on healthcare systems around the globe and caused a widespread disruption to medical education in general and to abroad electives in particular [32]. Due to stringent travel restrictions, limited cross-border mobility and university closures, oversea electives have been indefinitely placed on hold in many countries [33, 34]. Medical schools and other institutions switched face-to-face teaching from campus to virtual platforms [35]. As a corollary, the use of virtual methods for education and distant training greatly expanded [36, 37]. At the same time, many institutions closed their international elective programs for visiting students [38].

Park and Rhim emphasized that in April 2020 almost 70% of U.S. medical schools did not receive visiting students [38]. Subsequent to travel restrictions and the cessation of international visiting student programs, the number of abroad electives decreased substantially worldwide [39, 40]. In a British sample of 440 medical students, 77.3% (*n* = 340) had electives canceled or postponed [39]. An analysis of a German sample of students revealed a comparable picture [40]. Using retrospective analysis of two large elective databases, Egiz et al. reported that the number of short- and long-term international electives dropped significantly in 2020 [40].

**Fig. 2:**
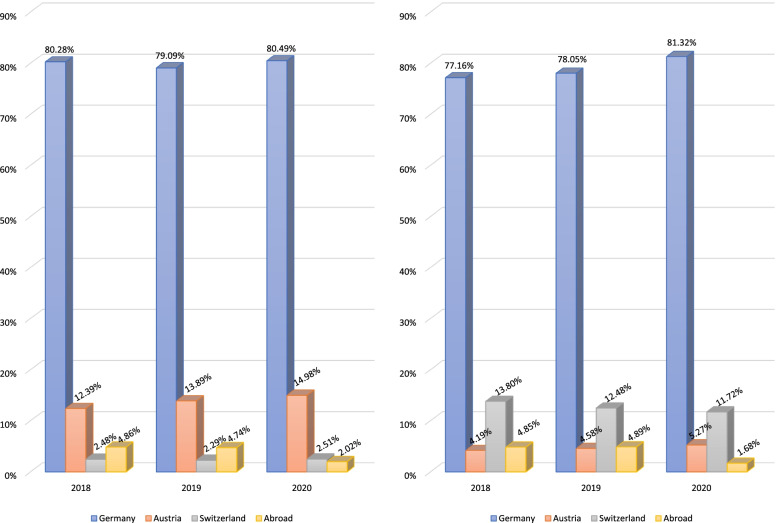
Number of abroad elective reports (outside of Germany, Austria and Switzerland) in a large German-based elective database cataloguing international elective testimonies. Left panel: short-term electives, right panel: long-term electives; modified from Egiz et al. [40]

**Fig. 3:**
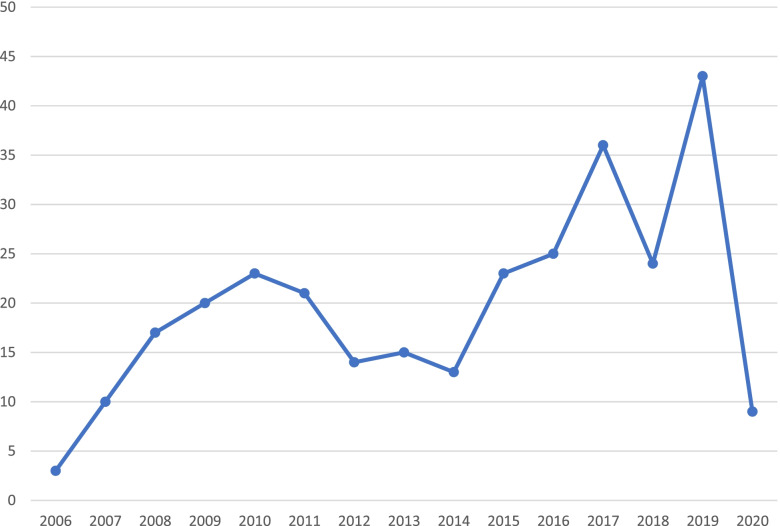
Number of abroad elective reports in Africa by medical students from German-speaking countries per year (2006–2020), modified from Storz et al. [42]

The authors observed an unparalleled drop in abroad electives by more than 50% for both elective types (Fig. 2) [40]. Institutional and logistic challenges in identifying sufficient clinical training sites for students required intense changes and prompt attention from medical educators [35, 41]. These are also reflected in another study by Storz et al., who investigated motivations for medical electives in Africa in Germany-based medical students [42]. The authors observed a sharp decline in international electives in Africa in 2020 (Fig. 3), and interpreted this as a proximate consequence of the COVID-19 pandemic [42]. A more global analysis by the same study group revealed a similar picture [43].

The trends observed in the aforementioned studies were frequently reinforced by students who shared their tertiary experiences on a more personal level [44–46]. The most prominent example was probably a British Medical Journal letter from a fourth year medical student who described how he lost half of his student selected components [44]. The pandemic thwarted long and meticulously-planned international electives and caused significant despair in students worldwide. This was reflected in another topical letter by Thundercliffe and Roberts who called the international medical elective “another victim to the pandemic” [47].

The future of the international medical elective is precarious and it is indeed questionable whether electives will return to their “former glory as a rite-of passage at the climax of undergraduate medical education” [47]. Although global health events, such as the COVID-19 pandemic or Ebola, constantly remind us of the need for international collaboration [48], it is likely that the pandemic is going to substantially change the abroad elective landscape in the next years.

### International electives in times of uncertainty and increasing disparities (2022 – pp.)

Although some experts suggested that the end of the pandemic is approaching [49], it is at present impossible to tell how long travel restrictions and other containment measures will be maintained. The unpredictable nature of travel restrictions will likely accompany medical students for a fairly long time. Electives, however, require long and extensive planning [50, 51]. A preparation time of 12 months or more is not uncommon for an abroad elective, depending on the destination and elective duration [52]. Many medical schools have stringent application requirements and long (often multi-step) application processes [52]. As long as a safe travel to the destination may not be guaranteed, many students will refrain from investing the time in extensive applications requiring numerous documents, certificates and official transcripts [43]. The same may apply to visa and scholarship applications.

In addition to that, elective application fees are usually non-refundable and may be as high as $1000 US Dollars per 4-week rotation [43]. Costs for medical electives is largely shoulder by medical students and apart from some very rare and competitive structured medical elective programs, students are responsible to pay the required fees, travel costs, daily life expenditures and accommodation on their own [50, 53, 54]. Thus, cost is often a crucial and defining factor for many students when planning electives. To make things worse, many students also lost their jobs and other revenue streams during the pandemic [40, 55]. The pandemic put a considerable number of students under financial strain [56], and many students will therefore think twice before submitting a costly application that in the end may be torpedoed by unexpectedly imposed travel restrictions.

The emergence of new virus variants is another unpredictable factor that warrants consideration in the elective planning process [47]. New contagious virus variants may require additional containment efforts [57], and could thus interfere with students’ plans. It is also likely that access to SARS-CoV-2- vaccines and vaccination programs will play a pivotal role in the peri- and post-pandemic medical elective. Several countries made vaccines for medical staff mandatory [58–60], and it is conceivable that medical students are going to require a certain number and combination of vaccinations to participate in a global elective in the near future.

This may bring along ethical dilemmas, as medical students from low- and middle income countries often have only limited access to vaccinations [61]. As such, these students will experience substantial difficulties when applying for an elective in a well-situated high-income country, where a certain combination of vaccinations could be mandatory to partake in a medical elective. This could be particularly problematic with mRNA-vaccines, which are still unavailable to citizens in many low-income nations [62]. In light of this realistic scenario, the pandemic is indirectly increasing global disparities and the so-called North–South gap previously described by Hanson et al. [63]. Thus, we project that within the next years, students from low-income countries will have even greater difficulties to secure an elective in a Western country, whereas vice-versa problems are rather unlikely to occur.

It is conceivable that the absence (or reduced number) of international elective students may also negatively affect host institutions (particularly in low-income countries). Multiple authors reported that host institutions and host preceptors may potentially benefit from visiting students in several ways [64–68]. On a professional level, international students may contribute to increased medical knowledge about disease processes, interpretation of diagnostic tests and professional exchange [64]. Visiting students may also be a valuable human resource contributing to patient care and providing practical assistance in wards, theatres and emergency departments. They may also strengthen the reputation of host institutions in the global community and provide opportunities for international collaboration [65, 67]. Most important, there are several indicators that international students bring financial benefits to host institutions, for example by paying elective fees or bringing donations, such as blankets, heaters and other equipment [65, 67, 68]. In light of the aforementioned points, it is not inconceivable that the pandemic-related halt of international electives negatively affected host institutions in several ways, although precise studies proving this hypothesis are still missing.

Disparities between students from low- and high income countries may also increase with regard to elective fees. Many hospitals and institutions depend on the revenue of collecting elective fees from international incoming medical students [42]. Elective fees may be subject to change upon re-opening of elective programs. To compensate for the lack of incoming international students within the last two years, host institutions are likely to increase their fees for visitors. Hosts will have to devote a great deal of energy to increased hygiene measures, regular staff testing and personal protective equipment organization as well as vaccination verification of incoming students. These additional financial and temporal expenditures may increase both administrative and elective fees. In a worst-case-scenario, post-pandemic electives could be a reserved privilege for well-situated students, whereas students with limited resources (often from low-income countries) will experience additional troubles when trying to secure an international medical elective.

The number of available elective placements in the upcoming years will not be comparable to pre-pandemic times. A gradual re-opening of visiting programs and policies that favor home-students will limit the number of elective positions for international visiting students in the near future. Thus, it is likely that for the remaining spots interstudent competition (which already increased during pandemic times [69]) is going to increase further. What many countries observed with regard to the residency seat-to-applicant ratio could also affect international medical electives [70]. Students with a stronger financial background that allows for “multi-applications” and a quicker compilation of application documents (and a faster transfer of the required fees) may have a crucial competitive advantage in this setting, whereas those from resource-limited countries may experience troubles to cover increased fees.

Online electives have been proposed as a promising alternative for face-to-face campus-based teaching, allowing medical students to partake in abroad electives remotely [71–74]. While electronic learning has been extensively investigated during the pandemic, it remains debatable whether this form of learning may compensate for in-person contact and learning abroad in a different cultural setting. Virtual electives lack hands-on experience and clinical examination skills [75]. Tele-courses also reduce the ability to actively (and personally) engage with learners and to provide personal feedback [76]. Both aspects, however, are essential to international electives. Interpersonal experiences in a different clinical, cultural, and resource contexts makes abroad electives special [16]. Whether this is achievable with an online-class remains debatable.

Ottinger et al. recently highlighted the importance of virtual education in a classical, non-international elective context [77]. Whether results are transferable to the abroad elective setting is unknown and has not yet been examined in clinical studies. Yet, many students engage in online electives because they have no other opportunities, and because they need letters of recommendation for residency applications [38, 78, 79].

Irrespective of the learning success of online electives, the classical sequence of organizing an international elective (information, application, preparation, implementation, de-briefing) is no longer the same [80]. This important (self-responsibility requiring) facet of abroad electives that once boosted students’ managing and organization skills is no longer required with online electives. Instead, students simply log-in into a prepared online module that limits personal interaction. It is self-evident that this is not the same as preparing for one of the main adventures of undergraduate medical education: an international elective.

Students who chose the second option will encounter countless problems related to the pandemic. Pre-departure trainings that help maximize the benefits and minimize elective related harms are largely cancelled at the moment due to the almost non-existent elective options [81, 82]. Elective testimonies and reports from former students, which often serve as a first-orientation, largely date back to pre-pandemic times [43].

As such, the COVID-19 pandemic represents a caesura for all those involved in international elective planning. This applies not only for students and host institutions, but also for researchers in the field. Research and literature about international medical elective is traditionally scarce [83, 84]. The pandemic further complicated on-site research and largely allowed for retrospective analyses and (systematic) reviews [4, 85]. Whether this is going to change within the next few years will mainly depend on the further course of the current pandemic.

There are many open questions to be address by future studies, which should closely investigate the elective landscape in post-pandemic times. One major focus should include (novel) barriers and obstacles to partake in electives after the pandemic. New studies may not only aim at investigating whether there are specific factors that may impede students from undertaking electives in the post-pandemic era (as projected in this review), but also look for potential approaches and solutions. Going abroad for international electives was hardly possible during the first two years of the pandemic, and this may also have deleterious consequences for global and public health education. A better understanding of these sequelae (and potential counter-measures) is urgently warranted. Finally, studies investigating whether and how virtual education affected international medical electives (if at all) would also be desirable.

## Conclusion

The COVID-19 pandemic substantially changed the international medical elective landscape, as oversea electives have been indefinitely placed on hold in many countries. Thus, the number of international electives decreased globally during the pandemic. A whole generation of medical students has been robbed of the opportunity to partake in international global health electives, which may affect global and public health education. The specific consequences of this development are yet unknown and subject to further research. Apart from well-investigated direct restrictions (such as the closure of international visiting student programs and travel restrictions) there are many other indirect problems and aspects that have received little attention during the last two years. Increasing disparities (for example a potentially worsening North–South gap) and ethical aspects that could arise once elective programs will gradually re-open will pose a substantial challenge to all those involved in international electives. The same may apply for potentially increasing elective fees and the administrative burden that is likely to be higher for prospective students partaking in electives. Given the tremendous importance of international electives for global and public health education, additional trials are urgently required to allow for a better understanding of pandemic-related sequelae in the abroad elective landscape.

## Data Availability

All data associated with this review will be made available from the corresponding author on reasonable request.
